# External Modulation Laser Module Assembly for Improving Measurement Performance of Homodyne Interferometry

**DOI:** 10.3390/s20195652

**Published:** 2020-10-02

**Authors:** Tao Zhang, Tao Sun, Jiean Li, Xingyu Zhao, Jiwen Cui

**Affiliations:** 1 Center of Ultra-Precision Optoelectronic Instrument Engineering, Harbin Institute of Technology, Harbin 150001, China; hitlja@163.com (J.L.); ZXY_IRIS_027@163.com (X.Z.); cuijiwen@hit.edu.cn (J.C.); 2 Space Environment Simulation Research Infrastructure, Harbin Institute of Technology, Harbin 150001, China; 3 Center for Precision Engineering, Harbin Institute of Technology, Harbin 150001, China; taosun@hit.edu.cn

**Keywords:** homodyne interferometry, noise suppression, laser source modulation

## Abstract

An external modulation laser module assembly (EMLMA) is proposed to suppress nonlinear errors in an interferometry system and improve its measurement performance. The EMLMA employs both phase modulation with radio frequency signal and a specific modulation amplitude switching mode, enabling the suppression of noise introduced by spurious reflections. The amplitude modulation reduces the influence of stray and background light by transforming the signal of interest to a high-frequency bandwidth. Experimental results show that the measurement error and stability of the interferometry system are significantly improved using the proposed light source module. After modulation, the spurious reflection-induced offset is decreased, and the measurement resolution improves from 7 to 2 nm. The EMLMA can replace the light source of any interferometric measurement system without altering the optical measurement structure. The proposed method reduces the influence of nonlinear errors in homodyne interferometry and provides a basis for further improvement of the interferometry performance.

## 1. Introduction

Laser interferometry is employed to realize rapid and ultra-precision displacement measurement in the fields of ultra-precision engineering and nanotechnology [1,2]. The development of these fields has set more stringent requirements on the measurement accuracy of laser interferometry [2,3]. Homodyne [4] and heterodyne [5] interferometry are the two most commonly employed techniques in the field of laser interferometry. The essential difference between them is the extraction method of the measurement information. Compared with heterodyne interferometry, homodyne interferometry has numerous advantages, such as no limit of speed measurement, a simple optical path, and effortless integration [6,7,8]. It moreover has obvious technical advantages and application prospects in numerous fields.

Homodyne interferometry obtains phase information through the periodic variation of the interference light intensity. Variations in the intensity of the measurement light have a large influence on the measurement results [4,6,9]; hence, homodyne interferometry is easily affected by changes in the external environment. Moreover, internal sources of uncertainty include nonideal optical and electronic elements, system installation errors, and laser power drift, as well as the DC offset error, unequal alternating current (AC) amplitude error, and nonorthogonal error [10,11,12,13], leading to nonlinear measurement errors exhibited in the measured displacement signal, generally ranging from the sub-nanometer to the tens of nanometers scale [10,11]. The nonlinear error generated in homodyne interferometry is one of the most important factors restricting further improvement of its measurement accuracy.

In recent years, numerous studies focused on reducing the influence of the nonlinear error in the interferometry system on the measurement results [14,15]. These studies mainly addressed the analysis of the error model, improvement of the system structure, and error compensation in the measurement signal.

The establishment of the nonlinear error model focuses on the nonideal error analysis of the polarizing prism, depolarizing prism, wave plate, and corner prism [10]. Moreover, there are many adjustable elements in the optical path of general laser interference, such that the nonlinear error caused by the inaccurate adjustment of the devices in the optical path is also one of the key research points [16]. By analyzing the influence of each nonideal element on the nonlinear error, the relationship between the position and attitude of each adjustable element and the error is given. In practical measurement, according to the analysis of the error model, the optical path structure must be optimized, and the nonideality of the optical elements and the adjustment deviation must be reduced as much as possible. The nonlinear error compensation method focuses on the follow-up signal processing. Only the DC offset error and unequal AC amplitude error can be corrected in real time [17]. The nonorthogonal error compensation algorithm is complex and time-consuming, such that it is difficult to achieve real-time compensation.

In the optical interferometry system, the nonlinear errors induced to the coherent noise arising from the spurious interference are very common, and much work has been done. For reducing the interference noise, the simple way is coating surfaces with antireflective coatings to disrupt reflections at the optical interface [18] or adjusting the measured surface to a certain angle to produce coherent cancellation [19]. However, these methods lead to the complexity of the preparation and adjustment of the optical system.

Another direct approach that prevents the interference noise is using a low temporal coherence illumination, such as the white-light [20,21] or the extended broadband LED [22]. However, it would give rise to a dispersion problem and require a certain experimental condition and adjustment method to generate the white-light signal/fringes compared with the laser-based systems. Some people proposed the phase error separation method in the phase shifting interferometry to reduce the interference noise [23,24]. For these methods, however, the derivation of the formula of coherent noise and phase error is complicated, and it can only be applied to the phase shifting interferometry system. Some interference measurement systems with special optical-path structure could also reduce or eliminate the interference noise to a certain extent [25], but these methods put forward strict restrictions on the optical system and cannot be applied to other interference measurement systems. In the measurement of Fizeau interferometer, the noise image caused by interference noise could be reduced or eliminated by processing of the interference image [26,27]. However, it is limited by the spatial frequency of the image. In addition, it is inevitable to change the interference signal while suppressing the coherent noise, resulting in the loss of some useful information.

On the other hand, the stray light, and intensity drift also affect the results of the interferometry system. Generally, the adjustment and optimization of the experimental device, such as adopting a filter or setting up a darkroom could prevent the stray light influence; adopting a laser source with higher stability or a constant temperature and humidity system could reduce the laser drift. At best, all these efforts are cumbersome and cost both time and money. In the field of signal communication, carrier modulation technology was widely used in the transmission and reception of slow changing signals or DC signals [28,29]. In the field of optical measurement, this technology has been evolved into lock-in technology [30,31], which shows excellent performance in weak signal detection. The lock-in technology is aimed at measuring the resulting signal, which is only embodied in signal processing at the signal receiving end. Meanwhile, the lock-in technology would increase the complexity of the signal processing part and reduce the speed of measurement to a certain extent. 

Nonlinear errors in the interferometry system can only be compensated and suppressed, whereas it is difficult to eliminate them completely. Conventional methods focus mostly on the optimization of the optical structure and elements, and on the subsequent signal processing and compensation, ignoring the possibility of improving the light source module to reduce the nonlinear error.

In this paper, we present an external modulation laser module assembly (EMLMA) based on hybrid phase/amplitude modulation to reduce the coherent noise arising from the spurious interference, stray light, and intensity drift. We introduce the phase/amplitude modulation method and the principle of the noise suppression, and we describe the system components of the EMLMA in detail. Further, we present the results of the experiment, which demonstrate the validity of the proposed method. The measurement performance of a grating interferometer was evaluated to determine the potential use of the proposed laser module in ultra-high-precision interferometry measurement system applications, and the conclusions are presented herein. Therefore, it can reduce the influence of nonlinear errors without affecting the structure of the original optical system.

## 2. Materials and Methods

### 2.1. System Design

In the homodyne interferometry system, the interference signal is inevitably affected by interference noise. As one of the main sources of interference noise, the spurious reflection light is caused by the reflection or multiple reflections of the light beam on the surface of nonideal transmission optical elements. When the spurious reflection light propagates in the system and interferes with the signal light, the spurious-reflection induced coherence offset (SICO) is formed. In general, the SICO will be directly superimposed on the interference measurement signal, thus changing its phase distribution, causing measurement errors and reducing the signal-to-noise ratio (SNR).

In this study, aiming at the characteristics of the additional optical path difference caused by the spurious reflection, the SICO could be eliminated by appropriately choosing the parameters of the phase modulation (PM) [32]. The expression of the light wave after PM is

(1) $$E_{0}(t)=A_{0}\cos\left[\omega_{0}t+\varphi_{0}+\varphi_{m}\cos(2\pi Ft)\right]$$

where *A*_0_ is the amplitude, *ω*_0_ is the angular frequency, and *φ*_0_ is the initial phase of the laser light, and *φ_m_* is the modulation depth and *F* is the modulation frequency of the modulator driving source.

The phase modulated beam is incident to the subsequent interference system for interference and detection. The intensity of the interference light is expressed as follows:

(2) $$I(t)={\left|E_{1}+E_{2}\right|}^{2}={\left|E_{1}\right|}^{2}+{\left|E_{2}\right|}^{2}+2\left|E_{1}\right|\left|E_{2}\right|\cos\left[\omega_{0}\frac{OPD}{c}+a\cos(2\pi Ft+\beta )\right]$$

where *OPD* is the optical path difference of two measurement beams, *c* is the speed of light, and *β* is the phase constant. *a* is the modulation amplitude of coherency to indicate the influence degree of coherency by PM, which is expressed as

(3) $$a=2\varphi_{m}\cdot \sin(2\pi F\frac{OPD}{c})$$

Then, by using the Jacobi–Anger identity, we can obtain the expression of interference light intensity:

(4) $$I(t)={\left|E_{1}\right|}^{2}+{\left|E_{2}\right|}^{2}+2\left|E_{1}\right|\left|E_{2}\right|\cos(\omega_{0}\frac{OPD}{c})\gamma$$

According to the coherence theory, the coherence coefficient *γ* is introduced to characterize the coherence ability of any two of the interfering beams and is expressed as follows:

(5) $$\gamma =J_{0}\left[2\varphi_{m}\sin(\pi F\cdot \frac{OPD}{c})\right]$$

where *J*_0_ is the zero-order baseband component of the first kind of Bessel function.

**Figure 1:**
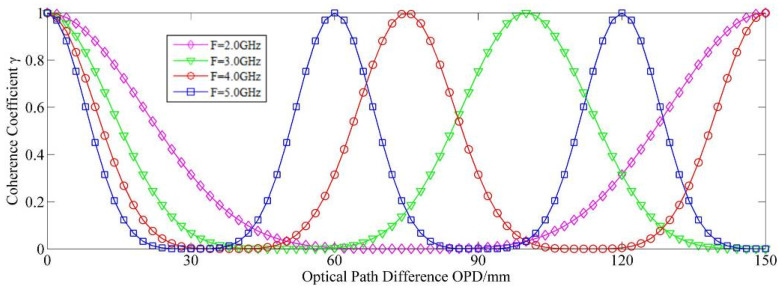
Coherence coefficient with respect to optical path difference at different modulation frequencies with 1.2 rad modulation depth.

The coherence coefficient γ characterizes the temporal coherence of the light source after PM, as shown in Figure 1. The curves represent the coherence coefficient of the light source with different optical path differences after PM. In comparison to the excellent temporal coherence of the laser source before PM, the temporal coherence coefficient of the laser source after PM changes periodically with the optical path difference between coherent beams, forming coherent peaks and valleys.

In the interference measurement system, the spurious reflection introduces an additional optical path difference relative to the original reference or measurement beams. By appropriately choosing the parameters of PM, the peak and valley positions of the coherence coefficient γ may be controlled and adjusted. For a given interferometry system, the coherence coefficient of the measurement and reference beams must be approximately 1, indicating no effect on the interference performance of the measured signal light. Meanwhile, the coherence coefficient of the measurement/reference beam and the spurious reflection light must be approximately zero, forming the coherent cancellation. Thus, the SICO can be effectively suppressed on the premise that the original interference signal is intact.

In the actual system, spurious reflection may occur at several positions. Therefore, the optical path difference introduced by the spurious reflection beam cannot be determined with precision, and consequently, the PM parameters cannot be accurately matched. Under the premise of constant modulation frequency, multiple modulation depths are continuously switched with a certain duty cycle to form alternating modulation depth.

Therefore, the synthesized coherent function *γ_s_* is expressed as

(6) $$\gamma_{s}=\sum_{i}d_{i}\cdot \gamma_{i}(\varphi_{mi}),$$

where *d_m_* is the time slot of the PM with *m*-th modulation depth and *γ_i_*(*φ_mi_*) is the coherence coefficient, in which *φ_mi_* denotes the modulation depth.

By fast switching of the PM depth, the effect is equivalent to the result of a summation of the separate contrast functions. In this study, the following parameters are used as input for Equation (6):

(7) $$\{\begin{array}{l} d_{i}=\frac{3}{16},\frac{5}{16},\frac{5}{16},\frac{3}{16}\;\mathrm{for}\;i=1,2,3,4\\ \varphi_{mi}=1.85,\;4.25,\;6.7,\;8.9\;\mathrm{for}\;i=1,2,3,4 \end{array}$$

**Figure 2:**
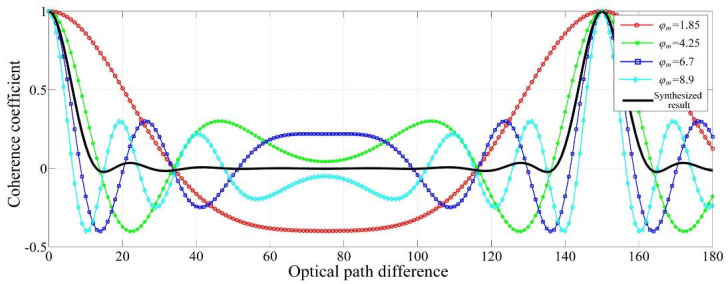
Synthesized coherent function as a function of the optical path difference.

The synthesized coherent function *γ_s_* is shown in Figure 2. The PM frequency of the light is 2 GHz. Figure 2 depicts the modulation result under the switching of multiple different modulation depths, to achieve the suppression of spurious reflections introducing interference noise across a large range.

### 2.2. Principle of Noise Suppression Based on Amplitude Modulation

Amplitude modulation (AM) of a laser source is performed to move the spectrum of the amplitude signal of light intensity to the high-frequency carrier frequency. Before the measured interference signals are processed by software, they are demodulated by the electronics, which moves the frequency components of the measurement signal near the carrier signal frequency back to the low-frequency position through the multiplier.

**Figure 3:**
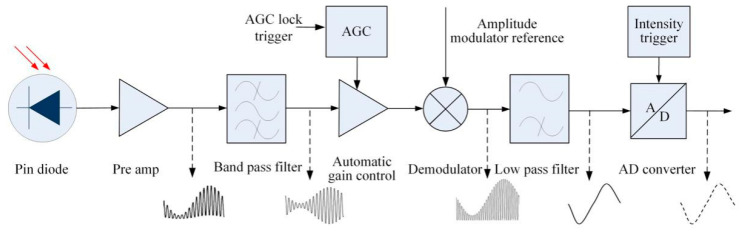
Schematic functional block and electrical layout of signal processing.

This means that the specific noise of the signal that originates from the path between modulation and demodulation effectively only contributes at the specific modulation frequency. This filters out noise and disturbances at other frequencies than the modulation frequency, especially in the low-frequency range. The schematic functional block and electrical layout of the signal processing is shown in Figure 3.

To reduce noise and crosstalk between the measurement beam and stray light, the signal is band pass filtered before it is normalized. The filtering is performed around the modulation frequency. The band pass removes the DC component in the signal, and the cross-talk is below 0.1% after the BP filter.

The normalized voltage/signal is multiplied by its synchronous carrier signal. The product is a measurement signal that includes an unwanted signal that is twice the carrier frequency. The phase of the carrier signal is matched to the phase of the measurement signal. Linear phase filters are used to filter out unwanted higher frequencies from the demodulated signal. At a frequency twice the cut-off frequency, the signal is attenuated (below 50 dB). Once the cut off frequency is set, the filters have a linear dependency of phase versus frequency. Thus, the time delay is constant.

Modulation technology increases the complexity of the system; however, it improves the working quality of the photoelectric system, contributes to signal processing in the transmission process, improves transmission capacity, and increases the SNR and measurement sensitivity.

In this study, the laser modulation induces the electronics to process the signals at frequencies of 40 kHz. If a higher frequency band is selected, the impedance of the capacitors decreases, which induces high leakage currents, and reduces the SNR. At lower modulated frequencies, the 1/*f* noise contribution becomes dominant.

### 2.3. EMLMA System

**Figure 4:**
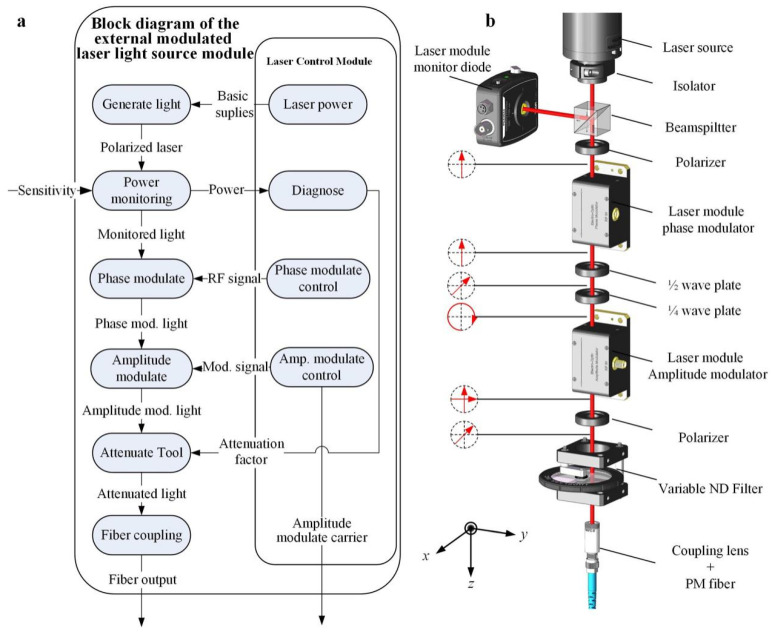
(**a**) Schematic functional block of external modulation laser module assembly (EMLMA). (**b**) Schematic of mechanical layout of EMLMA.

The schematic functional block and mechanical layout of EMLMA are shown in Figure 4.

The light source module of the EMLMA comprises the following parts:

(1) The first part is the coherent laser source. This study adopts a 21 MW He-Ne laser (Thorlabs HNL210LB) with a wavelength of 632.8 nm. The long-term power stability of the lasers is better than 2%. The short-term power stability is below 0.5 %. The beam diameter at the beam waist is 0.7 mm, such that it can pass through the aperture of the back-end modulator. The power is kept constant by adjusting the laser current. The function of optical isolator (Thorlabs IO-3D-633-VLP, USA) is to prevent the adverse effects of backward transmission light on light source and optical system.

(2) The second part of the light source module is the energy monitoring. The beamsplitter (BS) lets 95% of the light beam to pass through, while 5% of the energy is reflected to the side. The reflected beam energy can be detected by the photodetector to monitor the light intensity and stability of the laser. The splitting ratio of the beamsplitter and the sensitivity (in mW/Bit) of the photodetector must be calibrated. The intensity of the light beam entering the photoelectric modulator can be controlled according to the measured laser power at the photodetector.

(3) The third part is a radio-frequency (RF) PM unit containing a crystal (Newfocus 4431, USA). Applying a voltage across the electrodes of an electro-optic crystal changes its effective refractive index, and thus, the laser light that passes through the crystal can be modulated in phase by applying a modulated RF signal to the crystal. When a phase modulator is used, the laser beam must be well collimated, and its polarization must be oriented vertically to within 0.5° to ensure that the light wave undergoes a phase shift.

The temporal coherence of laser light can be reduced in a well-defined manner by special PM aiming at the characteristics of the additional optical path difference produced by the spurious reflection. By modulating the beam, the interference signal formed by the spurious reflection light is in the state of coherent cancellation, which thus does not affect the original interference measurement signal and effectively suppresses the spurious reflected interference noise.

In particular, the phase modulator must be controlled by the RF signal. The modulation voltage and frequency of the RF signal source is determined from the simulation results of spurious reflection induced OPD. Subsequently, the simulation results are used for the design and distribution design of the driving source of the phase modulator. The modules of the RF signal source, power amplifier, voltage-controlled attenuator, and square wave voltage-controlled signal are designed and manufactured accordingly. Finally, the modulator driver is used to drive the phase modulator.

(4) In the fourth part of the EMLMA, the phase modulated light passes through an amplitude modulator (Newfocus 4102). The electro-optic amplitude modulator can be considered as a voltage-tunable wave plate. We inserted a quarter-wave plate prior to the modulator, such that the input polarization state is circular. This eliminates the need for an electrical bias, which increases the usable input voltage to the entire range of the high-voltage amplifier. The applied voltage generates a variable phase delay between the two perpendicularly polarized components, corresponding to a change in polarization and leading to AM when passed through a polarizer.

The modulation frequency is 40 kHz. Modulation reduces the influence of stray and background light by converting the signals of interest to the high-frequency bandwidth. Different frequencies allow band filtering to reduce crosstalk between signals. After modulation, the modulation depth is larger than 97%.

(5) In the fifth part, a gray filter (Thorlabs NDM4, USA) is used to attenuate the modulated laser. The attenuation ranges from neutral density 0 to 4.0. The neutral density filter is controlled by manually varying the optical density by changing the angle of the filter in the optical path, which is used to assess the effect of reduced optical power on the electronics. The continuously variable reflective neutral density filters provide linear, adjustable attenuation within the coated region via rotation. The rotating axle has angular graduations that improve repeatability.

(6) The parallel laser beam is converged and coupled into a single-mode polarization-maintaining fiber (Thorlabs P1-630PM-FC-1, USA) with a core diameter of 4 μm through the fiber coupling lens (Thorlabs F280FC-B, USA). After transmission of the outgoing polarization beam through the fiber collimator, which is prealigned to collimate light from an FC/PC-terminated fiber with diffraction-limited performance, the parallel beam is formed for the subsequent optical interference measurement system.

As the light source module comprises numerous optical elements, a certain loss of energy occurs along the optical path. The beam power transmission performance of different elements of the light source module described in this study was measured, and the results are listed in Table 1. Notably, the parameters in this table depend on the selection of optical elements and are directly related to the installation and integration of the system. The transmission performance of the light energy in the light source module can be further improved by optimizing the system configuration integration.

## 3. Results

### 3.1. Experimental Setup

**Figure 5:**
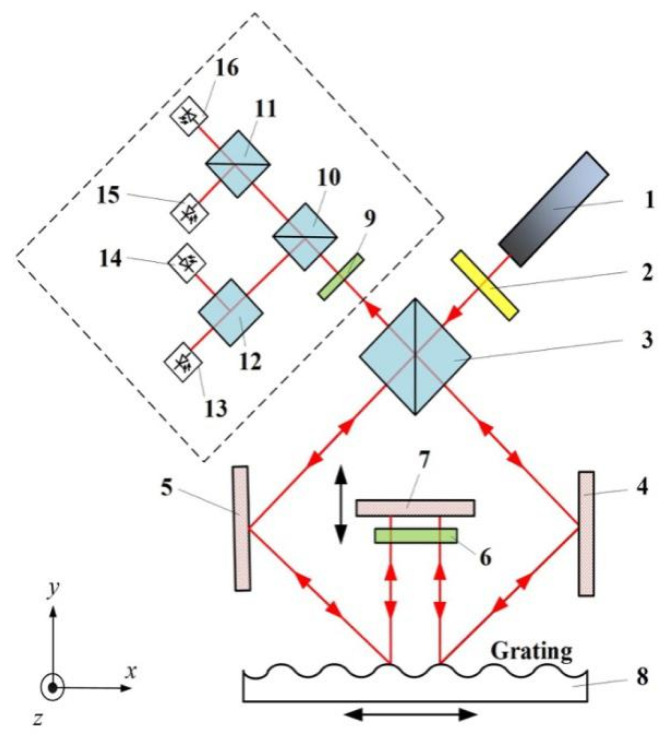
Grating interferometer system for evaluation of EMLMA performance. 1. Laser source; 2. 1/2 wave plate; 3,11,12. PBS; 4,5,7. Reflection mirrors; 6,9. 1/4 wave plate; 8. Measurement grating; 10. NPBS; 13,14,15,16. Photodetectors.

To evaluate the performance of the proposed light source module, a series of experiments was conducted on the interferometric measurement system, as shown in Figure 5.

The grating interferometer in Figure 5 is included in the homodyne quadrature laser interferometer configuration to correct the DC offset and unequal AC amplitude. The proposed EMLMA treats the laser light and transmits it to the optical system. The laser beam is split into P-polarized light and S-polarized light by the PBS (3), and the two resulting beams are incident to the reflective diffraction grating through mirrors (4) and (5), respectively. The +1st order and –1st order diffraction light of the two beams is vertically emitted from the grating surface. After the 1/4 wave plate (6), the beam is vertically incident to the mirror (7). The reflected beam is subsequently diffracted at the grating and returns to the original optical path. Two beams are combined to form interference signals through PBS (3), and four sinusoidal interference signals with a phase difference of 90° are obtained.

### 3.2. SICO Performance before and after PM

To verify the SICO in the interferometer system and differentiate the resulting interference, mirror 7 was moved along the y-axis instead of moving the grating in the measurement system along the *x*-axis.

In this case, there is no additional phase difference between the two measurement beams; hence, there will be ideally no change in the measured signal. However, the interferometer system is subject to SICO, which influences the measurement signal due to the interference effects of light reflected inside the prism/lens and light that is transmitted through the optical surface. The SICO effect is influenced by the distance to the optical surface, the surface type, and effectively results in measurement.

**Figure 6:**
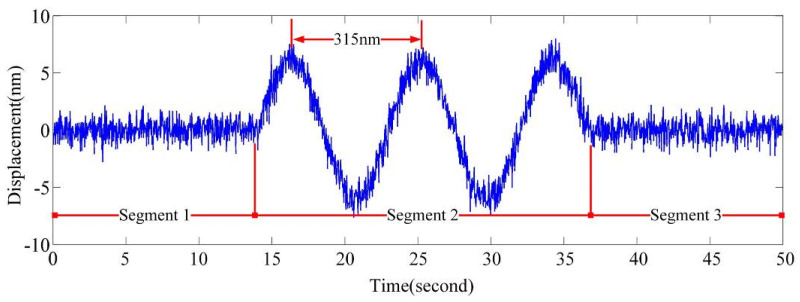
Experimental results of the interferometer for verifying the phase modulation (PM) performance of the EMLMA.

Figure 6 shows the measurement results of the interferometer. The first segment represents the result of the interference signal measurement without moving any optical element. Only a small amount of noise is contained in the measured signal.

In the second segment, we moved mirror 1 in the y-direction using a piezoelectric ceramic driver. At this time, the measured signal exhibits evident periodic variation with a period of approximately 315 nm. This corresponds to λ/2 of the adopted interferometer laser light (λ = 633 nm) and is recognized as the SICO.

In the third segment, mirror 1 continues its movement in the y-direction; however, when the PM is turned on, the periodic variation in the measurement signal disappears. The phase modulator operates by default to suppress interference effects in the measurement system. The SICO effects are reduced to the noise level by the PM for improved measurement performance.

We adopted the experimental method described above, because SICO is superimposed on the measurement results in the form of a phase error, and it is difficult to separate SICO completely from the conventional measurement signals using a conventional approach. Meanwhile, in the event that an angle deviation occurs in the adjustment of optical elements in the actual system, the mirror is not completely parallel to the grating. The displacement of the grating in the x-direction leads to a y-direction coupling displacement, which varies the distance between the optical surface and measurement grating, giving rise to the SICO phenomenon.

### 3.3. Stability Performance before and after AM

AM in the light source module is mainly used to improve the instability caused by stray light in the measurement system and suppress the influence of the drift of the light source on the measurement results, which reflects its immunity to environmental disturbances.

Therefore, we tested the stability of the experimental system. To this end, the system was enclosed in an insulating container to minimize air turbulence and temperature variations, and placed far from vibration sources, such as the upper computer case and signal generator.

**Figure 7:**
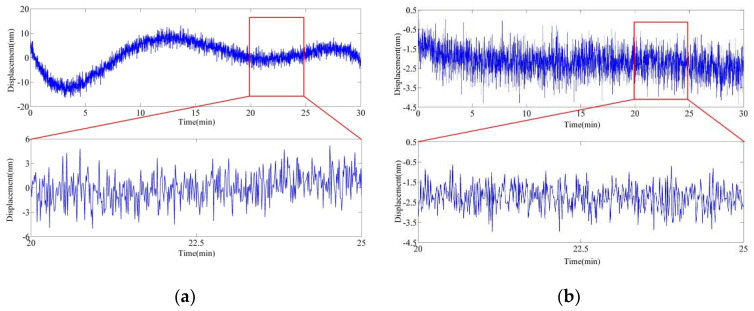
Experimental stability results on interferometer for evaluation of amplitude modulation (AM) performance in EMLMA. (**a**) Long- and short-term stability before AM. (**b**) Long- and short-term stability after AM.

First, the stability test was run without AM for 30 min, and the result is shown in Figure 7a. Subsequently, the stability test was run with AM for 30 min, and the result is shown in Figure 7b. In comparison to the measurement results in Figure 6, the stability of the system and the noise level are improved significantly after AM of the light source. The stability during 30 min is below than 5 nm displacement without any compensation, and the noise amplitude decreases significantly.

### 3.4. Displacement Resolution Performance before/after PM and AM

**Figure 8:**
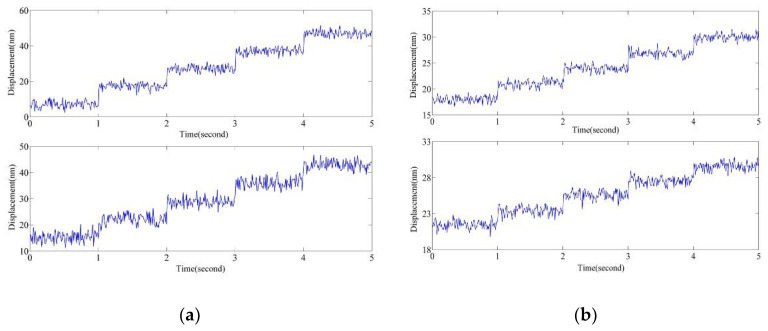
Experimental results of interferometer evaluating PM and AM performance of EMLMA. (**a**) Measurement resolution before the AM. (**b**) The measurement resolution after the AM.

To assess the measurement performance of the proposed absolute position measurement system, its measurement resolution was determined. In the displacement resolution experiment, the piezoelectric is driven to generate a step displacement, and the interference signal is output by the grating interferometer optical path. First, the experiment was conducted without AM and PM of the laser source. The piezoelectric ceramic driver executes steps of 10 and 7 nm, and the step interval is 1 s. The measurement results are shown in Figure 8a. Subsequently, the experiment was conducted with AM and PM of the laser source, as described in this study. Here, the piezoelectric ceramic driver executes steps of 3 and 2 nm, and the step interval time is 1s. The measurement results are shown in Figure 8b. When the step is 2 nm, it is still clearly distinguished in the measurement results with light source AM and PM. Thus, the resolution of the measurement system is improved from 7 to 2 nm after modulation of the light source.

## 4. Discussion

The analysis and comparison of these experimental results validate the theory proposed in this study. However, in practice, the nonlinear errors caused by nonideal factors in the interference system cannot be completely eliminated, neither by the proposed method, nor by the optimization of the optical system structure and error compensation for measured signals. The proposed EMLMA merely suppresses the SICO and noise induced by the stray light, background light, and intensity drift.

The PM method employed to suppress the measurement error caused by spurious reflection is particularly applicable in the equal-path interference system. This is because the PM periodically changes the temporal coherence of the light source. In the measurement system, the optical path difference between two interference paths must correspond to the position near the peak value in Equation (6) and Figure 2, to ensure that the measured interference signal has sufficient strength. In the theoretical analysis, the peak position of the coherence coefficient is periodically discrete, corresponding to the PM parameters. When the optical path difference between two interference paths of the interference system needs to change continuously (absolute distance interferometer or relative distance interferometer), this method is not applicable. However, the position of the zero optical path difference corresponds to the peak position of the coherence coefficient after PM, such that the PM method proposed in this paper is consistently applicable for the equal-path interferometry system.

If the optical path difference between the two interference arms of the interference system is not 0, but a certain value, the peak position of the coherence coefficient can be controlled artificially by the PM parameter so that the optical path difference corresponds to one coherent peak of the modulation curve. Therefore, the optical path difference of the interference signal is guaranteed to be near the peak of the coherence coefficient, as long as the optical path difference between the two interference paths of the adopted interferometry system is not zero, but a small change near a defined value (for example, the phase-shifting interferometry system). In this case, the method and EMLMA proposed in this paper are applicable. In contrast, the AM of the light source module substantially reduces the energy of the light source by half, which has certain impact on the measurement performance of the measurement system. However, owing to the PM/AM modulation of the light source module to suppress the error signal and noise, the measurement performance of the measurement system is improved under their comprehensive influence. The demodulation in the signal processing could influence the measurement speed of homodyne interferometry, this effect can be improved by improving the processing circuit module

## 5. Conclusions

The EMLMA proposed in this study was investigated for its ability to suppress the nonlinear error produced in the homodyne interferometry, such as SCIO, and the noise induced by stray light and intensity drift. Focusing on the characteristics of the optical path differences caused by spurious reflections within the interferometry system, the induced offset was eliminated by employing an appropriate PM, as the temporal coherence of the light source was periodically modulated. Meanwhile, the AM of the laser source operates by moving the spectrum of the amplitude signal of light intensity to the high-frequency carrier frequency, and the measured interference signals were processed by software. These were then demodulated by the electronics, which moved the frequency components of the measurement signal near the carrier signal frequency back to the low-frequency position through a multiplier and filter.

The system integration of EMLMA was realized in this study, and the elements were described in detail. The EMLMA module was used to evaluate the measurement performance of the grating interferometer before and after modulation. The experimental results show that the SICO and the stability of the measurement system were significantly improved by the proposed EMLMA module. The measurement resolution was improved from 7 to 2 nm before and after modulation. These results are consistent with the expectations of the improved overall performance of the interference-based metrology adopting the proposed EMLMA.

In comparison with the traditional method, an advantage of the proposed method is that the light source module is independent of the optical measurement structure. Thus, the EMLMA can be directly applied to different optical interference measurement systems. It shares similarities to other interferometric modalities in its noise suppression characteristics, but is free from complex mechanical systems required for path matching, algorithm or subsequent signal processing. Therefore, it can reduce the influence of nonlinear errors without affecting the structure of the original optical system.

The performance obtained in this study indicates that the theoretical methods and proposed EMLMA contribute to the reduction of the influence of nonlinear errors in homodyne interferometry and provide a basis for further improvement of the interferometry performance.

## Figures and Tables

**Table 1:** Beam power passing through each part of the EMLMA.

Element	Transmittance (%)	Optical Power Output (mW)
Laser	1	21
Faraday Isolator	0.95	19.95
1/2 Wave Plate	0.99	19.75
PBS	0.95	18.77
Phase Modulator	0.85	15.95
Amplitude Modulator	0.95	15.15
Polarizer	0.47	7.12
Attenuator	Adjust according to demand	To be confirmed
Fiber Coupling	0.65	4.63
Fiber Transmission Out of Fiber	0.9	4.17
